# Equine Umbilical Cord Serum Composition and Its Healing Effects in Equine Corneal Ulceration

**DOI:** 10.3389/fvets.2022.843744

**Published:** 2022-03-17

**Authors:** Xavier Peyrecave-Capo, Nathalie Saulnier, Stéphane Maddens, Bérengère Gremillet, Isabelle Desjardins

**Affiliations:** ^1^Equine Department, Faculty of Veterinary Medicine, VetAgro-Sup, University of Lyon, Marcy l'Etoile, France; ^2^Vetbiobank, Marcy l'Etoile, France; ^3^Department of Clinical Sciences, Veterinary Medicine, University of Liège, Sart-Tilman, Liège, Belgium

**Keywords:** horse (equus caballus), cornea, ulcer, umbilical cord serum, autologous serum, healing

## Abstract

**Background:**

Human autologous serum (AS) and umbilical cord serum (UCS) both contain growth and neurotrophic factors that promote corneal healing.

**Aim:**

Our objectives were to compare equine AS and UCS cytokine and growth factor profiles and to assess the safety and clinical feasibility of the therapeutic use of UCS eye drops in cases of spontaneous complex ulcers.

**Study Design:**

Prospective clinical trial.

**Methods:**

Vitamin A insulin growth factor, platelet-derived growth factor-BB, transforming growth factor (TGF)-β1 (enzyme-linked immunosorbent assay), interleukin (IL)-1β, IL-6, interferon-γ, and monocyte chemoattractant protein 1 concentrations were determined in 10 AS collected from different horses and 10 UCS sampled at delivery. Six client-owned horses presenting with complex non-healing corneal defects of >5 mm^2^ were included in a clinical trial and treated with conventional therapy and conditioned UCS drops for 8–15 days. Ulcer surface and time to complete epithelialization were recorded.

**Results:**

Median concentrations of vitamin A, insulin growth factor, and platelet-derived growth factor-BB were not significantly different in AS compared with UCS (respectively, 14.5 *vs*. 12.05 μg/ml; 107.8 *vs*. 107.3 pg/ml; and 369.1 *vs*. 924.2 pg/ml). TGF-β1 median concentration in UCS was significantly higher than in AS (3,245 *vs*. 2571pg/ml) (*p* = 0.04). IL-1β, IL-6, interferon-γ, and monocyte chemoattractant protein 1 concentrations were variable in AS and undetectable in UCS. The corneal median ulcerative area was 37.2 mm^2^ (6.28–57.14 mm^2^) and had a duration of 4–186 days (median 19 days). All lesions healed within 13–42 days (median 17 days). No adverse effects nor recurrences within 1 month were noticed.

**Limitations:**

The sample size was small. Spontaneous corneal epithelial defects presented with variable clinical characteristics. There were no age-matched control horses to assess corneal healing time and rate.

**Conclusion and Clinical Significance:**

Equine UCS may be beneficial, as it contains no pro-inflammatory cytokines and a greater concentration of TGF-β1 compared with AS. Topical UCS appears safe and may potentially be used as adjunctive therapy for equine complex non-healing ulcers.

## Introduction

Little is known about the healing kinetics of spontaneous corneal epithelial defects in horses. The wound healing response in the cornea involves an intrinsic cascade of autocrine and paracrine cytokine-mediated interactions between epithelial cells, stromal keratocytes, corneal nerves, and cells of the immune system (1). Corneal healing has been shown to be most rapid during the first 5 to 7 days, followed by a slower epithelialization phase (2). Simple ulcers usually heal quickly and without complication (3, 4), whereas complex ulcers are characterized by tardive healing (5). The deep layers of the stroma are affected, and a bacterial and/or fungal infection is usually present. Healing can be inhibited by stromal collagen lysis caused by lytic enzymes produced by microorganisms, inflammatory cells, corneal epithelial cells, and fibroblasts (6, 7). In this context, the use of topical biological therapies such as autologous serum (AS) in addition to conventional treatment has shown a clinical benefit in humans (8) and horses (5, 9).

Both AS and human umbilical cord serum (hUCS) contain many growth factors and neurotrophic cytokines in common with tears (10–12). Interestingly, concentrations of epithelial growth factor (EGF), transforming growth factor-beta1 (TGF-β1), and nerve growth factor are several folds higher in hUCS than in human AS (13–15). Furthermore, insulin growth factor (IGF-1) and vitamin A concentrations are higher in hUCS than in human tears (12). A recent study showed that compared with AS, hUCS had significantly lower concentrations of pro-inflammatory cytokines [interleukin (IL)-2, IL-6, interferon-γ (IFN-γ), and tumor necrosis factor-α] (16) but significantly higher concentrations of some anti-inflammatory cytokines (IL-4, IL-6, IL-10, and IL-13) (17). hUCS has a bacteriostatic effect thanks to antibacterial agents such as immunoglobulin G, lysozyme, and complement. hUCS has been demonstrated safe and effective for the treatment of severe dry eye syndrome and persistent epithelial defects in humans. In 2003, a controlled clinical trial showed that hUCS accelerates reepithelialization of persistent corneal epithelial lesions compared with AS (18).

To our knowledge, the composition and the ophthalmic use of equine umbilical cord serum (eUCS) have never been reported in horses. The objectives of this study were (1) to assess the composition of TGF-β1, platelet-derived growth factor (PDGF-BB), IGF-1, vitamin A, IL-1β, IL-6, IFN-γ, MCP-1, and IL-1Ra of eUCS in comparison with the equine autologous serum (eAS), (2) to assess the safety of eUCS topical administration in the treatment of equine ulcerative keratitis, and (3) to study the clinical feasibility of eUCS use in cases of complex corneal ulcers that do not heal using conventional treatments. We hypothesized that the concentration of trophic factors and anti-inflammatory cytokines would be greater in eUCS compared with eAS where local administration of eUCS would be well-tolerated and may possibly contribute to improving the corneal healing process.

## Materials and Methods

### Ethical Animal Research

The Animal Research Ethics Committee approved the study protocol (N°1923). Consent was given by owners or their representatives for the collection of peripheral blood and umbilical cord serum (UCS) before and horse inclusion in the clinical trial.

### Characterization of Serum Composition

#### Equine Umbilical Cord Serum Obtention

Ten healthy broodmares (7–18 years old) stationed in a prominent french stud farm were selected for this study. Chosen mares were prepared for foaling according to farm protocols, with adequate monitoring. Umbilical cord blood was collected immediately after foaling. The cord was clamped 6–8 cm from the umbilicus. Venipuncture of the umbilical vein was performed with a needle connected to a 50 mL syringe and transferred immediately into a clot-activator tube. The blood was shipped at ambient temperature to the laboratory and processed within 12–18 h after collection. Samples were processed under aseptic conditions. Blood was transferred into 50-ml tubes and centrifuged (2,500 × *g*/10 min). Serum was collected, filtered through a 0.22-μm pore filter membrane, and aliquoted into sterile single-use vials (1 ml/vial) for storage at −80°C until further use.

At the end of the processing, aliquots of serum were sent to an external control laboratory (Laboratoire de leptospires et analyses vétérinaires) for sterility testing (bacterial and fungal contamination). In addition, umbilical cord blood was submitted to a broad-spectrum polymerase chain reaction screening (Labéo Franck Duncombe) to confirm the absence of infectious agents. Twelve extraneous agents were tested, including *Babesia caballi, Theileria equi*, equine herpesviruses 1, 2, 4, and 5, *Rhodococcus equi, Anaplasma phagocytophilum, Borrelia burgdorferi, Leptospira spp*, equine arteritis virus, and *Mycoplasma* spp. as previously described (19).

#### Equine Autologous Serum Obtention

Ten healthy adult horses from the university teaching herd were included in the study (8 females and 2 geldings) aged 3 to 14 years (mean age 11 years old). Ten milliliters of blood was collected from the jugular vein into a syringe and transferred immediately into a clot-activator tube. Adult blood samples were processed similarly to umbilical cord blood. Briefly, samples were stored at room temperature for 12 h before centrifugation (2,500 × *g*/10 min). Serum was collected and aliquoted for storage at −80°C until further use.

#### Serum Analysis

Human ELISA kits were used to quantify transforming growth factor beta (TGF-β1), Platelet-derived growth factor (PDGF-BB), and insulin growth 5 factor (IGF) concentrations since cross-reactivity with horse serum has been previously validated (20). All ELISA kits were purchased from R&D Systems (TGF-B1 quantikine ELISA, PDGF-BB quantikine ELISA, IGF-I quantikine ELISA). An equine-specific ELISA assay was used to measure IL-1 receptor 117 antagonist (IL-1Ra) concentration (Thermo Fisher Scientific). All procedures were carried out in duplicate according to the manufacturer's instructions. Briefly, serum samples were diluted to appropriate concentration in dilution buffers (based on preliminary experiments) and processed according to manual recommendations. The plates were read on an ELISA microplate reader and analysed with SkanIt software (Thermo Scientific), using a four-parameter logistic curve. A customized horse 5-plex kit was designed to test for interleukin-1 (IL-1β), IL-6, IL-10, interferon gamma (IFN-γ), monocyte chemoattractant protein 1 (MCP1) (Merck Millipore). Briefly, samples or standards were mixed with antibody-linked magnetic beads and incubated with agitation on a plate shaker overnight at 2–8°C. Plates were washed 3 times and incubated with biotinylated detection antibodies, followed by Streptavidin-Phycoerythrin labelling. Following washes, samples were read on a Luminex^®^instrument with a lower bound of 24 beads per sample per cytokine. Each sample was measured in duplicate. If the coefficient of variation (CV) of duplicate wells was >20%, the sample was discarded from the analysis. Vitamin A was determined by ultra-high-performance liquid chromatography.

### Clinical Trial

Horses of different sexes, ages, and weights presented at the equine clinic from August 2016 to December 2017 with a diagnosis of ulcerative keratitis were considered for study inclusion. The study criteria included the presence of corneal ulceration of at least 5 mm^2^, a positive fluorescein staining, and hospitalization of at least 10 days, with a monthly follow-up after discharge. Eye perforating injuries, cases of ulcerations secondary to recurrent immune-mediated keratitis or equine recurrent uveitis were excluded. An explicit owner informed consent was obtained.

Medical history included ocular lesion duration, probable or known cause of the corneal lesion, treatments used before admission (including drugs, dosage, and duration), and clinical evolution. Data from the clinical examination were recorded on admission, including alertness, rectal temperature, hydration status, appetite, respiratory and heart rates, and body condition score (/9).

A complete ophthalmological examination was performed using a direct ophthalmoscope and/or a slit lamp. Auriculo-palpebral and supra-orbital blocks with subcutaneous lidocaine were used as needed, as well as sedation with intravenous administration of detomidine. Intraocular pressure measurement using a rebound tonometry device was done (Icare Tonovet, Icare Finland Oy; after topical tetracaine administration). Eye ultrasonography was carried out comparing both eyes. A standardized fluorescein test was carried out. Photography of the eye was taken with the camera held at a standardized distance under the same lighting conditions. A ruler was placed next to the eye for lesion measurements. Ulcerative areas and fluorescence intensity of the staining were measured using Image J software (version 1.47, National Institute of Health, Bethesda, MD, USA).

An ophthalmological score was calculated for each horse (Supplementary Data S1).

Ophthalmological examinations, fluorescein staining, intraocular pressure measurement, and eye photography were obtained every 48 h until resolution. Ocular ultrasonography was repeated between days 6 and 8 after admission.

Selected horses were previously treated with various combinations of topical antimicrobials, atropine, and with AS for two of them (Horses 1 and 6), which was stopped at least 7 days before admission. Horses were hospitalized and treated with conventional therapy (systemic flunixin, topical atropine, and antibiotics). Allogeneic UCS eye drops were instilled three times a day during an 8- to 15-day period. The total duration of eUCS therapy was determined by the clinical evolution or at the owner's request to discharge their horse from the hospital. In the hospital, eUCS eyedrop vials were kept either at −80 or −20°C if prepared within 2 weeks and, once opened, were kept at 4°C for a maximum of 48 h.

Size of the ulcerative area over time, fluorescence intensity, and time to complete corneal healing (i.e., negative fluorescein staining and absence of epithelial and stromal corneal loss) were recorded.

### Statistical Analysis

Statistical analyses were performed using Graphpad Prism (Graphpad software). If a growth factor concentration was below the limit of detection of the ELISA kit, the kit-specific threshold value was assigned to this sample to allow statistical analysis. Growth factor concentrations between adult and umbilical cord blood samples were compared by a Mann–Whitney *U* test, considering the small sample size. Statistical significance was set at *p*-values < 0.05. Due to the low numbers of clinical cases, this part of the study is descriptive, and statistical analysis was not done.

## Results

### Equine Umbilical Cord Serum Microbiological Analysis

Sterility tests confirmed the absence of bacterial and fungal contamination in eUCS samples used. Among the 12 microbiological agents tested, only *T. equi* was detected in one eUCS, then discarded the sample.

### Equine Autologous Serum and Equine Umbilical Cord Serum Characterization

**Figure 1:**
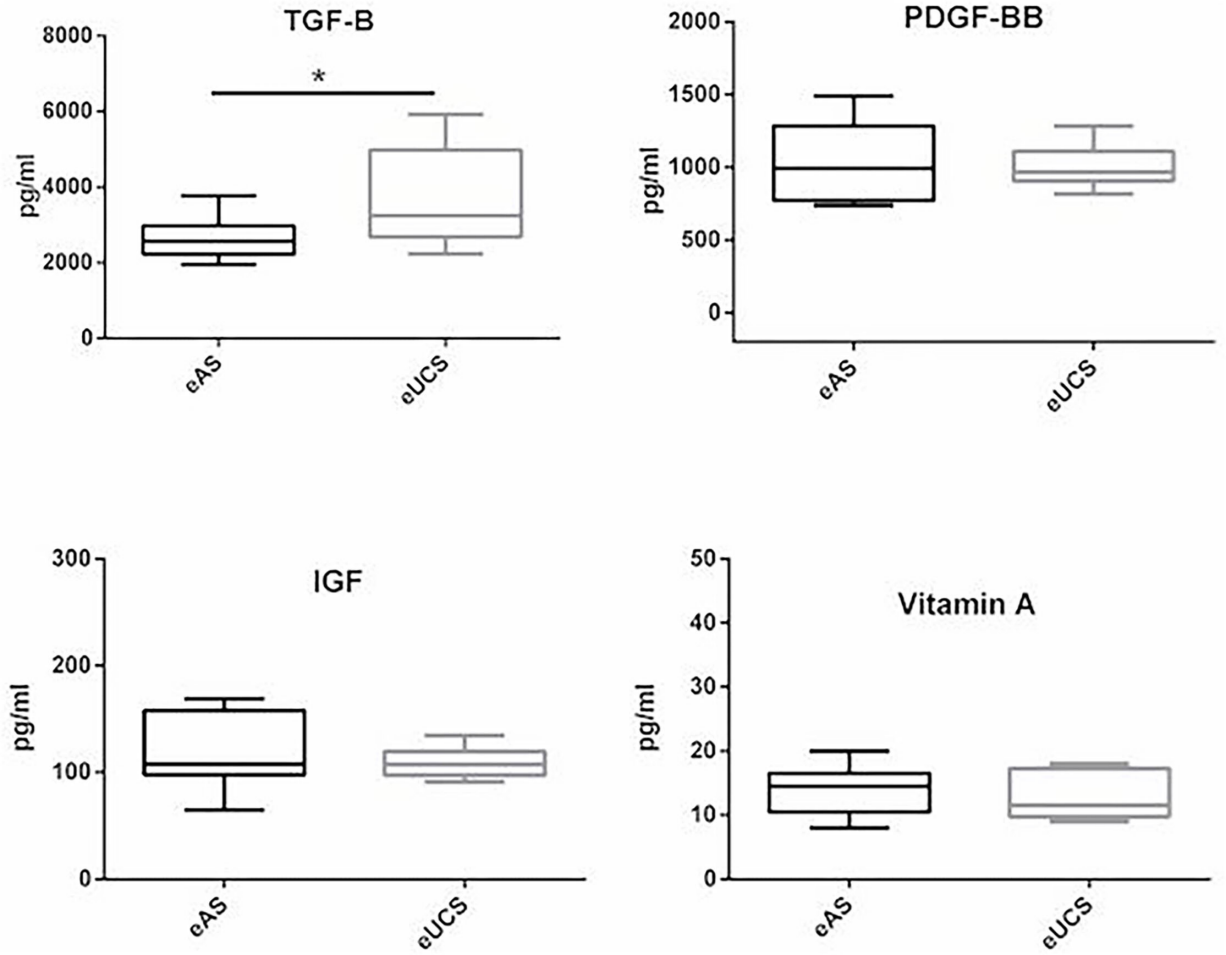
Boxplots of equine autologous serum (eAS) and allogenic equine umbilical cord serum (eUCS) concentrations in vitamin A and growth factors (PDGF-BB, TGF-β1, and IGF) (*n* = 10 samples for each group, except for PDGF-BB). ^*^Statistically significant, *p* = 0.04.

Growth factor concentrations of adult (*n* = 10) and umbilical cord (*n* = 10) serum were analyzed using ELISA assays. Data are shown in Figure 1. Median concentration of IGF was not significantly different in eAS [107.8 pg/ml; interquartile range (IQR), 97.8 to 157.9 pg/ml] and eUCS (107.3 pg/ml; IQR, 97.3 to 119.7 pg/ml). The PDGF-BB concentration was below the limit of detection of the kit in 5 of 10 adult samples and 3 of 10 umbilical cord samples. Excluding these samples from the analysis, median concentration of PDGF-BB was not statistically different in eUCS (*n* = 7) (967.5 pg/ml; IQR 905.8 to 1,110.3 pg/ml) and eAS (*n* = 5) (992.4 pg/mL; IQR, 771.9 to 1,281.4 pg/ml) (*p* = 0.61). In contrast, TGF-β1 median concentration in eUCS (3,245 pg/ml; IQR, 2,681 to 4,974 pg/ml) was significantly higher than in eAS (2,571 pg/ml; IQR, 2,223 to 2,976 pg/ml) (*p* = 0.04).

The concentration of IL-1Ra was measured in 10 eUCS with a median concentration of 643 pg/ml (IQR, 331 to 873 pg/ml), whereas IL-1Ra was determined in only three eAS samples with a median concentration of 13,645 pg/ml (IQR, 9,580 to 15,000 pg/ml).

Cytokines concentrations of adult (*n* = 3) and umbilical cord (*n* = 10) serum were analyzed using multiplex assays. Although the eUCS concentrations of IL-1β, IL-6, IFN-γ, and MCP1 were below the limit of detection (provided by the manufacturer, respectively, 26 pg/ml for IL-1β; 0.6 pg/ml for IL-6; 41 pg/ml for IFN-γ; 208 pg/ml for MCP1), heterogeneous results were observed for the eAS samples. Among the three donors, IL-1β concentrations ranged from 81 to 5,712 pg/ml, IL-6 concentrations ranged from 0 to 3,864 pg/ml, IFN-γ concentrations scaled from 0 to 23,993 pg/ml, and MCP1 concentrations ranged from 1,095 to 2,536 pg/ml.

Vitamin A results showed no significant difference between eUCS samples (median concentration: 11.5 μg/dl, IQR, 9.75 to 17.25 μg/dl) and eAS (median concentration: 14.5 μg/dl, IQR, 10.5 to 16.5 μg.dl) (*p* = 0.52).

### Clinical Trial

**Figure 2:**
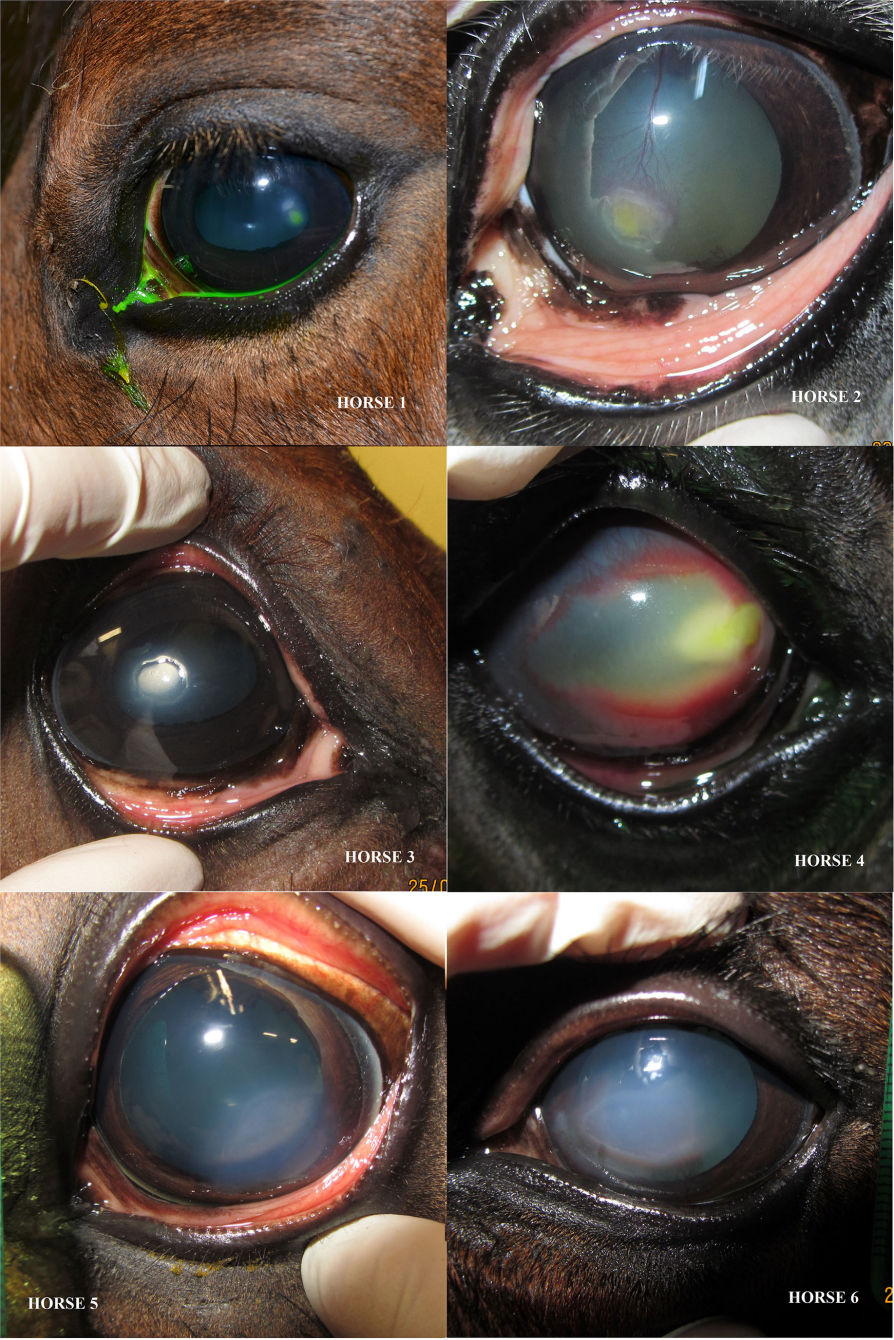
Images of eyes of six cases on admission showing various clinical presentations of complex ulcers in six cases.

**Figure 3:**
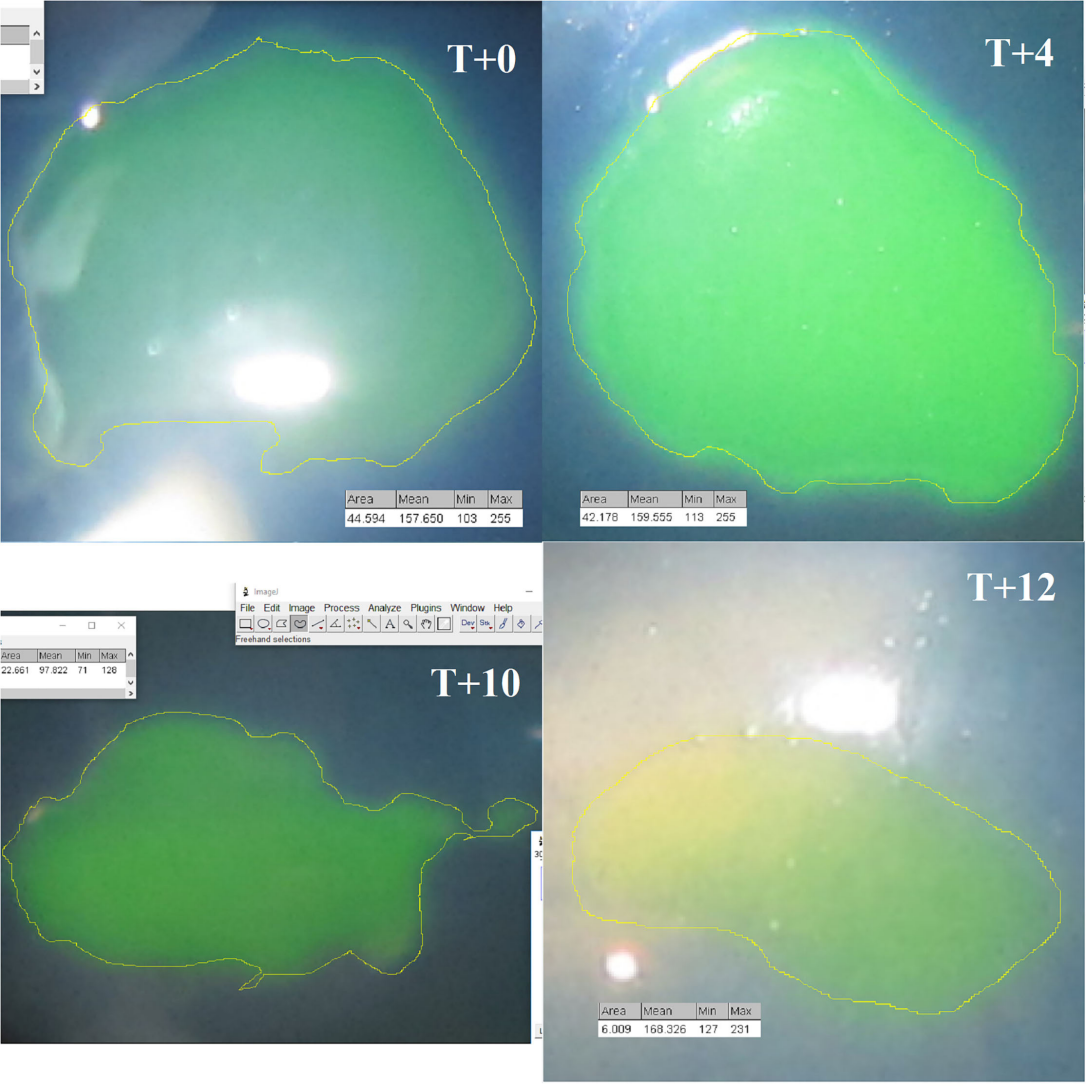
Decrease in ulcerated area in horse 5 at T0, T + 4, T + 10, and T + 12 days, using ImageJ software.

Six horses of various breeds (1 pony, 1 thoroughbred, 3 French saddle horses, and 1 Arabian) were included in the study (Table 1). Five were mares, and one was a male. The median age was 4.5 years (range 4 months to 17 years). The ulcerative lesion was detected in the left eye in four cases and in the right eye for two horses. Median corneal ulceration duration before admission was 19 days, ranging from 4 to 186 days. All horses presented with a complex ulcer. For three of them, the ulcerative lesion was irregular, whitish, and raised epithelial edges. One horse was diagnosed with a stromal abscess secondary to deep epithelial loss. Also finally, two of them presented with non-inflammatory, indolent superficial lesions (Figure 2). Using direct eye measurement, eye photography, and Image J area calculation, the surface of corneal loss was calculated between 6.28 and 57.14 mm^2^, with a median surface of 37.2 mm^2^ (Figure 3).

**Table 1:** Clinical data for the six included horses.

**Horse**	**1**	**2**	**3**	**4**	**5**	**6**
Age	1.5 years old	17 years old	1.5 years old	4 months old	13 years old	7 years old
Gender	Mare	Mare	Mare	Male	Mare	Mare
Breed	French saddle horse	French saddle horse	Arabian	Pony	French saddle horse	Thoroughbred
Affected eye	left	left	right	right	left	left
Ulcer duration (days)	186	12	16	8	4	49
Ulcerated area (mm^2^)	6.28	26.4	40.8	33.6	57.14	47.32
Ulcer depth	Epithelial superficial	Epithelial superficial	Epithelial superficial	Mid stromal	Epithelial superficial	Epithelial superficial
Ulcer margin	Normal	Whitish and slightly raised	Whitish and slightly raised	Yellowish and raised	Whitish and raised	Whitish and raised
Neovascularization on admission	None	Moderate	None	Severe	Very slight	Moderate
Subpalpebral lavage catheter	No	Yes	Yes	Yes	Yes	No
Previous medical therapy before admission	Topical -atropine -AM^†^ (norfloxacin+rifampicin) -vitamin B12 -N-acetyl cysteine -cyclosporine A - autologous serum (60 days TID ^‡^, stopped 42 days before eUCS § therapy) Systemic NSAIDs (flunixin)	Topical -atropine, -AM^†^ (tobramycin+rifampicin) -iodine (once) Systemic NSAIDs (firocoxib)	Topical -AM^†^ (framycetin) -corticosteroids (10 days)	Topical -atropine -AM^†^ (neomycin+polymyxin B) -N-acetyl cysteine Systemic NSAIDs (flunixin)	Topical -atropine -AM^†^ (neomycin+polymyxin B)	Topical -atropine, -AM^†^ (tobramycin) -sodium hyaluronate - autologous serum (10 days BID ^‡^, stopped 7 days before eUCS therapy)
Associated eye lesions before EUCS therapy	None	Moderate anterior uveitis	None	Stromal abscess Moderate anterior uveitis	Moderate keratitis	Moderate keratitis
Cytological analysis of corneal scrapping	Normal	Suppurated inflammation without bacteria or fungi	No	No	No	Yes
Microbiological analysis	Negative	Negative bacteriologic exam	No	No	No	Yes
Other medications used during EUCS therapy	None	Topical -atropine, -AM^†^ (tobramycin+rifampicin) -iodine (once) Systemic NSAIDs (flunixin)	Topical -atropine, -AM^†^ (tobramycin) Systemic NSAIDs (flunixin)	Topical -atropine, -AM^†^ (ciprofloxacin +rifampicin) -iodine (once) Systemic NSAIDs (flunixin)	Topical -atropine -AM^†^ (neomycin+polymyxin B) Systemic NSAIDs (flunixin)	Topical -atropine, -AM^†^ (ciprofloxacin) Systemic NSAIDs (flunixin)
EUCS therapy duration (days)	15	8	12	12	10	8
Time for complete healing (days)	13	14	15	15	42	22
Mean healed area/day (mm^2/^day)	0.48	1.88	2.72	2.24	1.36	2.15
Corneal ulceration recurrence on the affected eye	No	No	No	No	No	No
Complications on the affected eye	None No corneal scarring	Temporary moderate keratitis	Slight temporary non ulcerative keratitis Granuloma eyelid (subpalpebral lavage system insertion)	Temporary anterior uveitis Corneal scaring	No	Temporary keratitis
Other systemic diseases	None	PPID ^♦^ Chronic uveitis on the contralateral eye	Septic jugular thrombophlebitis	*Rhodococcus equi* pneumonia Septic jugular thrombophlebitis	Acute colic just before the eye ulceration – foreleg laminitis	Fungal ulcerative keratitis, stromal abscess, and then recurrent uveitis on the contralateral eye

*AM^†^, antimicrobials*;

*PPID ^♦^, Pituitary Pars Intermedia Dysfunction*;

*BID ^‡^, twice a day; TID ^‡^, three times a day*.

All included cases were treated with systemic flunixin, topical atropine, and antimicrobials. A subpalpebral lavage system was used when the eye was too painful (four cases). Two drops of eUCS were applied topically three times a day, even if horses had a subpalpebral lavage system, to avoid eUCS stagnation in the lavage system and possible mixture with other medications. The serum was administrated between 8 and 15 days, depending on the clinical evolution (median duration: 11 days). No adverse local or systemic effects were noticed, such as acute conjunctivitis and scleritis, palpebral edema, corneal superinfection, fever or urticaria.

Complete corneal healing was defined by a negative fluorescein staining and the absence of corneal epithelial loss seen on ophthalmic examination. Every corneal lesion resolved properly. Three horses healed during the course of eUCS administration, and three horses healed partially during eUCS therapy. Complete reepithelialization was achieved, respectively, at 11, 24, and 32 days after the eUCS therapy was stopped, whereas those three horses were still treated with the previous conventional therapy. The median resolution time was 17 days (range: 13 to 42 days). The median daily healing kinetic was calculated using the following formula: the surface of the ulcerative area before eUCS therapy/days to achieve complete healing. This rate was assessed between 0.48 and 2.72 mm^2^/day (median: 2.01 mm^2^/day). If needed, mechanical debridement of the ulcer margins was performed during eUCS therapy and afterward if healing was incomplete at the end of the 2 weeks of eUCS therapy.

Global ophthalmic scoring, blepharospasm, corneal edema decreased over time during eUCS therapy and until healing. In two cases with intense neovascularization, a decrease in the vessel's intensity and the surface was seen between 2 and 5 days of therapy. On the contrary, for the two horses for which no vessels were present, neovascularization developed within 3 to 4 days of eUCS application.

No recurrence of corneal ulceration was noticed in the month after complete healing.

Two horses had no ophthalmic sequelae. A slight to moderate temporary keratitis was noticed in three horses and a little persistent anterior uveitis in one case.

## Discussion

The major findings of the study were that 1—eUCS contains a significant concentration of several growth factors involved in the resolution of corneal defects (IGF, TGF-β1, and PDGF), whereas it is devoid of pro-inflammatory cytokines (IL-1β, IL-6, IFN-γ, and MCP1); 2—the procedure is safe, as no adverse effects have been seen; and 3—the naturally occurring ulcers treated with eUCS for the six included horses healed.

### Equine Umbilical Cord Serum Characterization

In the present study, cytokine and growth factor profiles were examined in eUCS for the first time and compared with adult serum. First of all, it has been shown that vitamin A and growth factor concentrations remain stable in AS, which has been frozen at −20°C for 3 to 6 months (21), so, in our study, those molecules would have been well-preserved.

Our results showed similar amounts of IGF, PDG-BB, and vitamin A in eAS and eUCS, whereas a significantly higher concentration of TGF-β1 was measured in eUCS compared with eAS. It is important to note that the low sample size could impact the non-statistically results, and more samples would be necessary to ensure that a proper conclusion may be drawn.

TGF isoforms [predominantly TGF-β1 (21)] are present in human tear fluids and confer important proliferative effects of the epithelium (22, 23). PDGF-BB stimulates equine corneal epithelial cell and keratocyte proliferation *in vitro* in horses and in rabbits (24–26). IGF is a neurotrophic factor promoting corneal epithelial wound healing through different mechanisms (27–29). It has been successfully used in humans to help resolve corneal epithelial lesions (30, 31). Vitamin A is important in maintaining the health of the ocular surface and necessary for the proliferation, differentiation, and maturation of healthy epithelial cells (32). Vitamin A deficiency has been associated with keratomalacia in humans (33, 34).

To date, no information is available about the growth factor content in eUCS, whereas human UCS has been characterized. Interestingly, Yoon et al. reported comparable results with ours in their study evaluating human AS and human UCS composition (30). Higher levels of EGF and TGF-β were detected in UCS, whereas the concentrations of vitamin A and IGF were lower in UCS compared with AS. Another study reported higher levels of EGF, TGF-β, nerve growth factor, and vascular endothelial growth factor in human UCS compared with other blood-derived preparations (35). Together, these data suggest that human and equine UCS express a common neurotrophic growth factor profile with some peculiarities for each species. In our study, we reported a similar concentration of vitamin A in adult and umbilical serum samples (11.5 pg/ml in eUCS and 14.5 pg/ml in eAS), whereas levels of vitamin A in human AS and UCS have been shown to be statistically different (21, 30, 36). Nonetheless, median levels of vitamin A in human and equine serum samples are significantly different, with a concentration of 1.000-fold higher in human samples. Vitamin A content of equine tear film has not been assayed yet, but it has been described that vitamin A content in human UCS is higher than natural tears (14).

In our study, we detected variable concentrations of pro-inflammatory cytokines among the different eAS. Pro-inflammatory cytokines in AS may reflect an effective inflammatory process against infection even in a non-symptomatic animal. Using autologous AS extemporaneously prepared as a therapeutic agent prevents the evaluation of cytokine content in the sample before use and thereby may alter clinical efficacy. The presence of pro-inflammatory factors may contribute to corneal epithelial defect maintenance. Conversely, in eUCS, which allows biochemical characterization before use, we did not detect any pro-inflammatory cytokines, as recently reported in human UCS (16). Interestingly, albeit no IL-1β has been measured in eUCS, we detected some level of IL-1Ra. However, the clinical significance of this finding remains unknown. IL-1Ra is an anti-inflammatory cytokine. IL-1a, IL-1, and IL-1Ra have been detected in the human corneal epithelium (37) and tears (38). Moreover, IL-1Ra therapy can significantly decrease corneal inflammation and lead to enhanced corneal transparency after traumatic damage (39–41).

As a whole, low levels of pro-inflammatory cytokines and significant expression of growth factors in eUCS could represent a more beneficial therapeutic combination to hasten resolution and justify evaluation in larger clinical trials.

### Equine Umbilical Cord Serum for Ophthalmic Use: A Safe Approach

In our pilot study, no adverse reaction was detected in horses after the application of eUCS and during the follow-up of the patient.

Allogeneic blood may pose infectious risks, but these can be drastically minimized through careful donor selection and testing based on risk assessment of microbiological exposure of the products and epidemiological considerations (19). Ideally, a quarantine period may ensure the highest level of safety for preventing infectious disease, but this is difficult to implement in the field. In humans, umbilical cord blood has been proposed to carry a lower risk of carrying an infectious agent than the paired maternal peripheral blood. We also highlighted this privileged status of equine umbilical cord blood and umbilical cord tissue in a previous study (29). In our study, *T. equi* was detected in one umbilical cord blood sample and discarded. Clearly, extensive microbiological screening is not practical with extemporaneously prepared autologous products, and indeed, various latent pathogens are prevalent, including equine herpesvirus, known to be widespread in asymptomatic horses (29). No one can predict the potential adverse effect of a microbiological agent transplanted into a new ecosystem.

Therefore, compared with AS, for which no infectious testing for extraneous agents nor sterility testing is performed, a standardized preparation of eUCS is much clearly safer.

Focusing on immunological concerns, eUCS are acellular products, therefore, offer little or no risk of patient alloimmunization due to donor red blood cells, leucocyte, or platelet antigens, nor is there a relevant risk of cell-mediated immune reactions initiated by donor cells against patients' tissues (42). In addition, eUCS does not contain immunoglobulins.

### Clinical Feasibility Study

In several species, spontaneous chronic corneal epithelial defects have been defined as corneal defects that fail to heal despite appropriate therapy (43). The recurrent erosion syndrome in humans is characterized by a non-adherent epithelium surrounding the corneal ulcer (44–46). In horses, non-healing corneal ulcers, also known as indolent ulcers or chronic corneal epithelial erosions, are quite common (37, 47, 48). Among the well-known causes of non-healing ulcers is dry eye syndrome, which is prevalent in humans but not in horses (5, 12, 38). Recurrent trauma, foreign bodies, and corneal dystrophies are persistent sources of irritation or re-ulceration, and treatment with corticosteroids, old age, and pituitary *pars intermedia* dysfunction also constitute significant etiologies (49–51).

In our pilot clinical study, all horses eventually healed, and corneal healing was achieved at the end of the eUCS therapy for half the cases; although for three horses, conventional therapy was prolonged for 11 to 32 days after eUCS was stopped. Horses 1 and 6, exhibiting the longest history of corneal epithelial defect, had been previously treated topically with AS without success, for 10 and 60 days, BID to TID, respectively (and was stopped 7 and 42 days, respectively, before initiating eUCS therapy). hUCS eyedrops have shown beneficial effects in treating recurrent and persistent corneal defects in humans associated with several primary conditions such as dry eye syndrome, neuropathic keratophathies, or idiopathic non-healing ulcers (15, 18, 30, 52–55) and were more effective compared with hAS (56). Considering our cases, healing times and rates were variable among horses. In comparison with simple recent ulcers, complex ulcers heal slowly. Surgically induced corneal defects in horses have been estimated to heal at 0.6 mm per day (57). Very few *in vivo* studies have indeed reported a healing rate in equines. Corneal healing of epithelial defects has been shown to be most rapid during the first 5 to 7 days, followed by slower epithelialization attributed to exhaustion of surrounding epithelial cells or changes in the underlying corneal stroma (2).

eUCS's growth factors could stimulate angiogenesis, cell proliferation, and migration and could reduce collagenase activity during this second phase (58). In humans, anti-proteases such as α2-macroglobulin are higher in hUCS compared with peripheral blood serum (11).

No abnormal opaque corneal scaring was observed after complete healing in the six horses treated with eUCS containing a high concentration of TGF-β1. It would have been interesting to quantify TGF-β3 in our eUCS samples because TGF-β3 has been shown to alleviate scar formation in the stroma and is considered to have anti-fibrotic activity (29, 59).

As no recurrence of ulcerative lesions was noticed the following month, it seems that the newly attached epithelium provided an adequate adherence for the underlying stroma.

The use here, of an allogeneic blood product instead of an autologous one, was well-tolerated. A recent crossover study with autologous and allogeneic serum eye drops for the management of ocular surface disease in humans suggests that both alternatives have comparable efficacy and tolerability (60).

In this pilot study, eUCS was administrated neat. In humans, AS eyedrops are diluted (final concentration 20%) before use. The rationale for diluting the serum is to decrease TGF-β levels to physiological concentration to limit its antiproliferative activity, which may suppress and promote stromal fibrosis. Nonetheless, this also reduces the concentration of trophic factors, potentially limiting beneficial effects. Furthermore, some authors have reported successful clinical outcomes with undiluted serum without any detrimental effects (61).

### Limits of the Study

Assessing the surface of a corneal defect may be challenging and should be standardized as much as possible. We normalized the measurements of the corneal lesion, as described in a previous *in vivo* study (2), by the use of a single-lens reflex camera set at a fixed focal length with imaging software, but nevertheless, the irregular shape of the defect may, however, create a bias.

An important limitation of the study reported here was the small sample size and diverse characteristics of the corneal epithelial defects regarding ulcer size, deepness, and duration of occurrence before inclusion/hospitalization. We attempted to minimize these effects by including only complex ulcers while standardizing the conventional ocular therapy and clinical follow-up. Moreover, the horses' ages were heterogeneous, and old age inevitably can slow corneal healing (50–52).

Another major limitation is the absence of a control group to compare eUCS efficacy with conventional therapy with eAS or placebo. We decided to assess the eUCS effects on naturally occurring corneal ulcers in client-owned horses, insisting on treatment and not a placebo. It would have been difficult to match cases with identical criteria regarding age, ulcer duration, ulcer surface, and deepness, but a control group receiving either AS or placebo would be essential to compare clinical efficacy in corneal healing. Surgically induced defects in experimental horses with healthy corneas would have probably shown a higher healing rate compared with spontaneous and complex ulcerations.

Eventually, the frequency of eUCS administration was empirically decided. Higher periodicity as described in human trials affected with dry eye syndrome (62) would have to be considered in a larger blinded randomized control study. Moreover, the duration of eUCS therapy was variable according to the clinical evolution or at the owner's request to discharge their horse from the hospital.

In conclusion, our study is the first to report the clinical use of eUCS for complex corneal ulcer management in horses. eUCS therapy has several advantages compared with other blood products. It is easily accessible, available in large quantities, and can be prepared in advance, allowing characterization of a consistent product before releasing it for therapeutic use. It is also much less likely to contain pathogens and, most importantly, does not incite an inflammatory and immune response when applied to the ocular surface. eUCS contains a significant amount of soluble proliferative and immunomodulatory factors compared with eAS that have the potential to hasten corneal reepithelialization. Further studies are warranted to determine whether equine allogenic UCS can hasten corneal reepithelialization compared with AS or conventional therapy alone.

## Data Availability Statement

The raw data supporting the conclusions of this article will be made available by the authors, without undue reservation.

## Ethics Statement

The animal study was reviewed and approved by VetAgro Sup Animal Research Ethics Committee. Written informed consent was obtained from the owners for the participation of their animals in this study.

## Author Contributions

XP-C, NS, SM, BG, and ID participate to the conception and design the study and to acquisition, analysis, and interpretation of data. XP-C, NS, SM, and ID participate to drafting and revising the article critically for important intellectual content. All authors have approved the version to be published.

## Conflict of Interest

SM and NS are employees and shareholders of Vetbiobank at the time of the submission for publication. The remaining authors declare that the research was conducted in the absence of any commercial or financial relationships that could be construed as a potential conflict of interest.

## Publisher's Note

All claims expressed in this article are solely those of the authors and do not necessarily represent those of their affiliated organizations, or those of the publisher, the editors and the reviewers. Any product that may be evaluated in this article, or claim that may be made by its manufacturer, is not guaranteed or endorsed by the publisher.
