# Supplementary material for: Equine Umbilical Cord Serum Composition and Its Healing Effects in Equine Corneal Ulceration
Source: Front Vet Sci. 2022 Mar 17;9:843744. doi: 10.3389/fvets.2022.843744 (PMC8970184; doi:10.3389/fvets.2022.843744)
Supplement: Supplementary file 1 [file Data_Sheet_1.PDF]

DATA S1: Ophthalmological scoring.

Ophthalmologic Parameter	Response	Score RIGHT	Score LEFT
Blepharospasm intensity	ABSENT: Eyelids are fully open in normal light conditions; cilia perpendicular to the ground surface	0	0
	SLIGHT: Eyelid less than half closed; slightly down cilia	1	1
	MODERATE: Eyelids more than half closed, down cilia	2	2
	SEVERE: Eyelids fully closed, vertical cilia	3	3
Epiphora intensity	ABSENT: No traces of ocular secretion	0	0
	SLIGHT: Appreciable amount of secretion is present in the nasal canthus of the eye, overflowing when pressing	1	1
	MODERATE: continuous secretion flowing from the nasal canthus of the eye	2	2
	SEVERE: continuous secretion flowing from the nasal and temporal canthus of the eye	3	3
Epiphora nature	ABSENT: No traces of ocular secretion	0	0
	SEROUS: Transparent secretion	1	1
	MUCOPURULENT WHITISH: White thick secretion	2	2
	MUCUPURULENT YELLOWISH: Yellow to brownish thick secretion	3	3
Conjunctivitis	ABSENT: pink pale conjunctival mucosa ; no perilimbal inflammation	0	0
	SLIGHT: flushed reddish conjunctiva ; some perilimbal inflammation	1	1
	MODERATE: bright red bulbar & palpebral conjunctiva ; moderate perilimbal inflammation	2	2
	SEVERE: dark beefy red bulbar & palpebral conjunctiva ; congestion; +/- petechia	3	3
Scleritis	ABSENT: whitish sclera and thin vessels	0	0
	SLIGHT: whitish sclera and distended vessels	1	1
	MODERATE: Pink to red sclera + distended vessels	2	2
	SEVERE: Reddish sclera + large amount of distended vessels +/- petechia - hemorrhage	3	3
Corneal edema intensity	ABSENT: fully transparent cornea	0	0
	SLIGHT: Some loss of corneal transparency- underlying structures clearly visible with light	1	1
	MODERATE: Some loss of corneal transparency – homogeneous white stroma - underlying structures barely visible with light	2	2
	SEVERE: Complete loss of corneal transparency - homogeneous white stroma - underlying structures cannot be visualized with light	3	3
Corneal edema localization	ABSENT: no edema present	0	0
	FOCAL: edema restricted to the corneal ulcerative lesion	1	1
	REGIONAL: edema extending around the ulcerative lesion	2	2
	DIFFUSE: edema in a large portion of the cornea	3	3

Ophthalmologic Parameter	Response	Score RIGHT	Score LEFT
Neovascularization intensity	ABSENT: no neovascularization	0	0
	SLIGHT: thin vessels of less than 2mm	1	1
	MODERATE: vessels of more than 2mm, mostly thin – “brush” appearance	2	2
	SEVERE: vessels easily seen - reddish appearance of the cornea – big vessels surrounded by thin numerous “ brush” vessels	3	3
Neovascularization localization	ABSENT: no neovascularization	0	0
	FOCAL: vessels restricted to less than 25% of the limbus, from only one side, next to the lesion	1	1
	REGIONAL: vessels present between 25 and 50% of the limbus, or from more than one side	2	2
	DIFFUSE: vessels diffusely present all around the limbus	3	3
Myosis (affected side)	ABSENT	0	0
	PRESENT	2	2
Corneal ulcer	ABSENT: Negative fluorescein testing / no epithelial loss visible without staining	0	0
	PRESENT: Positive fluorescein testing / epithelial loss visible without staining	5	5
Corneal ulcer evolution	DECREASED: Ulcer area is smaller than the last control (>than 5% decrease)	0	0
	STABLE: Ulcer area unchanged compared to the last control (<than 5% area increase or decrease)	2	2
	INCREASED: Ulcer area is bigger compared to the last control (>than 5% area increase).	4	4
Hyphema	ABSENT: no blood or hematoma visible in the anterior chamber (direct ophthalmoscopy)	0	0
	PRESENT: blood or hematoma visible in the anterior chamber (direct ophthalmoscopy)	2	2
Hypopion	ABSENT: no pus or whitish material seen in the anterior chamber (direct ophthalmoscopy)	0	0
	PRESENT: pus or whitish material seen in the lower part of the anterior chamber (direct ophthalmoscopy)	2	2
Synechia	ABSENT: no synechia, normal shape of the pupil	0	0
	PRESENT: synechia seen – abnormal pupil shape	2	2
Cataract	ABSENT	0	0
	PRESENT	2	2
Intra-ocular pressure With sedation (detomidine) and regional blocks	NORMAL: 17 - 28 mmHg or < 20% difference with normal eye.	0	0
	DECREASED: < 17 mmHg or <20% lower compared to the contralateral healthy eye	2	2
	INCREASED: > 28 mmHg or >20% higher compared to the contralateral healthy eye	4	4
TOTAL SCORE		/50	/50
